# *Arabidopsis* Mitochondrial Voltage-Dependent Anion Channels Are Involved in Maintaining Reactive Oxygen Species Homeostasis, Oxidative and Salt Stress Tolerance in Yeast

**DOI:** 10.3389/fpls.2020.00050

**Published:** 2020-02-28

**Authors:** Sibaji K. Sanyal, Poonam Kanwar, Joel Lars Fernandes, Swati Mahiwal, Akhilesh K. Yadav, Harsha Samtani, Ashish K. Srivastava, Penna Suprasanna, Girdhar K. Pandey

**Affiliations:** ^1^ Department of Plant Molecular Biology, University of Delhi South Campus, New Delhi, India; ^2^ Nuclear Agriculture and Biotechnology Division, Bhabha Atomic Research Centre, Mumbai, India

**Keywords:** Por1, mitochondria, reactive oxygen species, stress, mitochondrial membrane potential, *Arabidopsis*

## Abstract

Voltage-dependent anion channels (VDACs) are conserved proteins of the mitochondria. We have functionally compared *Arabidopsis* VDACs using *Saccharomyces cerevisiae Δpor1* and M3 yeast system. VDAC (1, 2, and 4) were able to restore *Δpor1* growth in elevated temperature, in oxidative and salt stresses, whereas VDAC3 only partially rescued *Δpor1* in these conditions. The ectopic expression of *VDAC* (1, 2, 3, and 4) in mutant yeast recapitulated the mitochondrial membrane potential thus, enabled it to maintain reactive oxygen species homeostasis. Overexpression of these VDACs (*AtVDACs)* in M3 strain did not display any synergistic or antagonistic activity with the native yeast VDAC1 (ScVDAC1). Collectively, our data suggest that *Arabidopsis* VDACs are involved in regulating respiration, reactive oxygen species homeostasis, and stress tolerance in yeast.

## Introduction 

Voltage-dependent anion channels (VDACs) are highly conserved protein, present in all eukaryotic species examined so far (Colombini, 2012). These are thought to regulate metabolite transport between mitochondria and the cytoplasm in both physiological and pathological conditions (Kroemer et al., 2007). VDACs are β-barrel proteins of a single subunit of the molecular mass of 30 kDa. They possess a conserved structure as well as similar biophysical properties across species (Colombini et al., 1996). In yeast, two genes encode VDAC isoforms (*ScVDAC1* and *ScVDAC2*) whereas in mammals, including mice and human have three isoforms (Kusano et al., 2009). The knockout of the major VDAC gene *ScVDAC1* renders the mutant yeast (commonly referred to as *Δpor1*) hypersensitive to grow in non-fermentable carbon source like glycerol due to impaired respiration (Dihanich et al., 1987; Lee et al., 1998; Graham and Craigen, 2004).

Complementation of this mutant by heterologous VDAC proteins has been widely used to identify functions of VDACs in different organisms (Blachly-Dyson et al., 1997; Rohl et al., 1999; Komarov et al., 2004; Wandrey et al., 2004; Desai et al., 2006; De Pinto et al., 2010). *ScVDAC2* could complement *Δpor1* when expressed under the *ScVDAC1* promoter or when multiple copies of *ScVDAC2* were present (Blachly-Dyson et al., 1997). The *Drosophila melanogaster* genome has four VDACs and complementation studies showed that two proteins, DVDAC and CG17137 were able to rescue *Δpor1* growth defect. Subsequent experiments proved both proteins had channel forming ability, indicating that even proteins having partial similarity to VDAC primary sequence can still retain the essential functions typical to a VDAC (Komarov et al., 2004). The human VDAC family has also been extensively characterized by using the *Δpor1* strain. Summarizing the collective data from different reports on complementation experiments, we can infer that—(a) the human *VDAC1* and *VDAC2* can complement *Δpor1* better than human *VDAC3* and this property is due to the N-terminus of human VDACs (Blachly-Dyson et al., 1993; De Pinto et al., 2010; Reina et al., 2010) and, (b) this difference in VDAC3 structure again gives it a selective advantage to protect *Δpor1* from hydrogen peroxide (H_2_O_2_) better than VDAC1 and VDAC2 (Karachitos et al., 2016). Similar to the human counterpart, the mouse *VDAC3* also was predicted to have a different function than mouse *VDAC1* and *VDAC2* through complementation experiments in *Δpor1* (Sampson et al., 1997).

Even in plant system, such as potato, wheat, lotus, pearl millet and rice; the *Δpor1* strain has been used to assess the complementation capability of VDACs (Heins et al., 1994; Elkeles et al., 1997; Wandrey et al., 2004; Desai et al., 2006; Godbole et al., 2011). Characterizing the VDAC genes in *Arabidopsis* is vital in the context of plant science as the information from the model plant can often be used to construct a hypothesis of the protein function in higher plants. Like typical VDAC, the *Arabidopsis* VDACs majorly localizes into the mitochondria (Tateda et al., 2011). So far, the *Arabidopsis* VDACs have been implicated in both plant growth and biotic and abiotic stress responses (Lee et al., 2009; Yan et al., 2009; Tateda et al., 2011; Robert et al., 2012). These studies have concentrated mostly on examining the loss-of-function mutants to interpret the results (Lee et al., 2009; Tateda et al., 2011; Robert et al., 2012). Though the animal VDACs are well studied for their role in reactive oxygen species (ROS) homeostasis, very little is known about plant VDACs (Csordas and Hajnoczky, 2009; Reina et al., 2010). So, we have characterized the *Arabidopsis* VDAC proteins using *Δpor1* to test their role in response to ROS and salt stress inducing agents. Using this system, we demonstrate the role of *Arabidopsis* VDAC proteins in respiration, maintaining mitochondrial membrane potential (MMP), ROS homeostasis, and (oxidative and salt) stress.

## Materials and Methods

### Plant Material, Stress Treatment, and Expression Analysis

The Colombia-0 (Col-0) ecotype was used for real-time PCR analysis. Plant growth and RNA isolation were done according to Sanyal et al. (2017). Salt, cold, ABA, and drought treatment were performed according to Kim et al. (2003). For methyl viologen (MV) treatment seedlings were treated hydroponically in half strength MS media spiked with 20 μM MV and control samples were kept in the same MS media for the duration of the treatment without MV. *Pseudomonas syringae* pv. tomato (Pst) was grown overnight in Kings broth at 28°C and were resuspended in 10 mM MgCl_2_ to 1 × 10^5^ cfu ml^-1^. This was used to infect 4 weeks old adult plants using a needleless syringe and they were then incubated to the indicated time period and tissue was collected and frozen in liquid nitrogen for RNA isolation. For qPCR analysis, primers were manually designed and are listed in **Supplementary Table 1**. Quantitative real-time PCR reaction was carried out in ABI Prism 7000 sequence detection system (Applied Biosystems, USA) using KAPA SYBR FAST Master Mix (KAPABIOSYSTEMS, USA). *ACTIN2* was used as an endogenous control to normalize the cDNA variance among the samples. Relative expression was computed by Y = 2^Ct^, where Y is a relative expression and Ct is the difference between a particular *VDAC* and *Actin2* under that particular condition (Wang et al., 2015). The experiment was performed with three independent biological replicates and each was measured with three technical replicates. The statistical significance of the differences was assessed using a Student's *t* test.

### Yeast Strains and Growth Conditions

Wild type *Saccharomyces cerevisiae* strain M3 (*MATa lys2 his4 trp1 ade2 leu2 ura3*) and M22-2 also known as *Δpor1* (*ScVDAC1* knockout yeast), derived from M3 contains a deletion of most of the POR1 coding region due to insertion of the yeast LEU2 gene at this locus. Yeast strains were grown on rich medium YP (1% yeast extract, 2% peptone) supplemented with 2% glucose (YPD) or 3% glycerol (YPG) and 2% agar was added for solid plates at pH 5.5. The plates were incubated at 30°C.

### Cloning and Expression of *Arabidopsis* Voltage-Dependent Anion Channels in Yeast

To analyze and compare the possible effect of different *Arabidopsis* VDAC isoforms in complementation of *Δpor1* mutant yeast cells, the coding regions of all the VDACs were PCR amplified. The amplified products were cloned in pGV8 vector (Agarwal et al., 2003) under the control of GPD constitutive promoter to yield constructs pGV8-VDAC1, pGV8-VDAC2, pGV8-VDAC3, and pGV8-VDAC4. Resulting constructs were confirmed by sequencing. The sequences of primers used are listed in **Supplementary Table 2**. For yeast transformation, plasmid DNA containing different isoforms of *Arabidopsis* VDAC were linearized with *Apa*I. The linearized constructs were separately introduced into a wild type yeast strain M3 and *Δpor1* using PEG/lithium-acetate transformation (Sanyal et al., 2017). Empty pGV8 vector was also transformed in both strain to be used as a control. Transformants were selected on agar plates containing 2% glucose in yeast nitrogen base supplemented with essential nutrients except for uracil. Resulting transformants in *Δpor1* mutant background were named as (*Δpor1*)pGV8VDAC1, (*Δpor1*)pGV8VDAC2, (*Δpor1*)pGV8VDAC3 and (*Δpor1*)pGV8VDAC4. Similarly, transformants created in M3 background were named (M3)pGV8VDAC1, (M3)pGV8VDAC2, (M3)pGV8VDAC3 and (M3)pGV8VDAC4. The expression of AtVDAC proteins in yeast (M3 and *Δpor1* strains) was verified by immunoblotting, using the anti-AtVDAC1 antibody (AS07 212, Agrisera) (1:5000). Yeast mitochondria were prepared according to Leggio et al. (2018). Anti-Mtg2p was used as a mitochondrial marker (1:2000) (Datta et al., 2005).

### Cell Viability Assay and Growth Kinetics

The relative growth of the transformants was estimated by spotting 10 fold serial dilutions (10^-2^ to 10^-4^) of the cells on the surface of YPD and YPG plates, incubating the plates at 30°C and 37°C and examining the plates daily for up to the indicated days. The growth kinetics experiment was performed according to Magri et al. (2016) with three independent biological replicates. For acetic acid sensitivity assay, transformants cells were dotted on YPD plates containing 75 mM acetic acid. For MV treatment YPD plates supplemented with 3 mM MV were used. For salt stress, transformed cells were dotted on YPG plates supplemented with either 1M KCl or 1 M NaCl plates.

### Detection of Mitochondrial Membrane Potential and Reactive Oxygen Species by Confocal Microscopy

The MMP was detected using confocal microscopy by using the fluorescent probe 2-[4(dimethylamino) styryl]-1- methylpyridinium iodide (DASPMI) according to Reina et al. (2010). Dihydrorhodamine 123 (DHR 123) was used for the detection of ROS according to Reina et al. (2010). The confocal visualization was performed using the Leica confocal SP5 microscope (Germany). DHR 123 and DASPMI were excited using 515 and 488 nm lasers, respectively and the emission wavelengths were set at 525–575 and 560–630 nm, respectively.

### Quantification of Mitochondrial Membrane Potential and Reactive Oxygen Species by Flow Cytometry

MMP was quantified by flow cytometry, using DASPMI as fluorescence probe (Magri et al., 2016). Yeast samples were grown in 3% YPG overnight for a primary culture followed by a secondary culture under constant shaking at 30°C. DASPMI (5 μg/ml of culture) was added to the tubes bearing secondary culture. Fluorescence of yeast cells (50,000 per sample) was analyzed by flow cytometry analysis using a BD FACSCalibur™. The data were analyzed using BD CellQuest™ Pro. The unstained M3pGV8 and *Δpor1*pGV8 were used as reference samples for relative quantification. Each experiment was repeated thrice in triplicate with independent biological replicates. Data were statistically analyzed by Student's t-test. A value of P < 0.001 was taken as significant.

Intracellular ROS content was quantified by flow cytometry using dye DHR 123 with some modifications (Magri et al, 2016). Yeast samples were grown overnight in 3% YPG for a primary culture under constant shaking at 30°C. The secondary culture was set under constant shaking at 30°C for 6 h. DHR 123 (5 μg/ml of culture) was added to the secondary culture. Same flow cytometer instrument that was used to measure DASPMI uptake was used to measure DHR123 uptake. Each experiment was repeated thrice in triplicate with independent biological replicates. Both DHR123 and DASPMI were excited using the 488 nm laser and detected using FL1 and FL2 filter, respectively by the flow cytometer. Data were statistically analyzed by Student's t-test. A value of P < 0.001 was taken as significant.

## Results

### *Arabidopsis* Voltage-Dependent Anion Channels Exhibits Differential Expression Pattern in *Arabidopsis*

**Figure 1:**
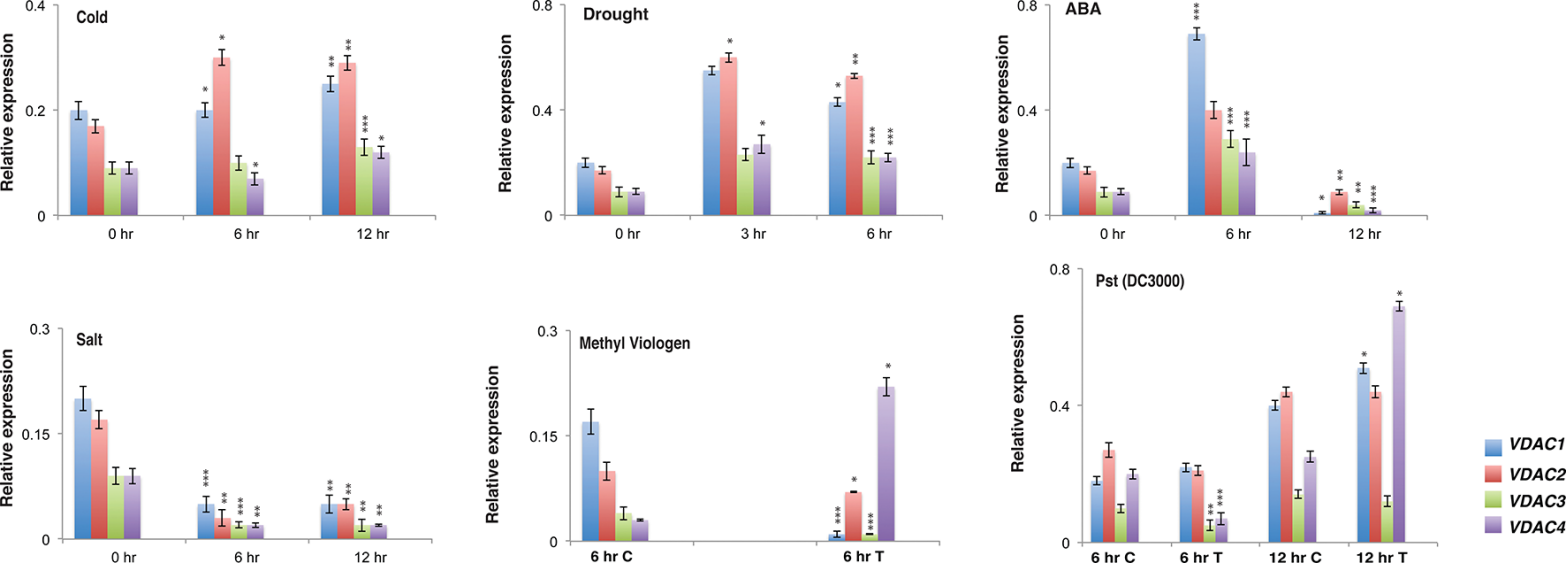
Expression analysis of different *Arabidopsis* VDACs under different stress stimuli. Relative expression of different *VDAC* family members under abiotic and biotic stress. RNA was isolated from stress treated {cold, drought, ABA, salt, Pst (DC3000) and MV} Col-0 samples followed by quantitative real-time PCR. The treatment and quantitative real-time PCR procedures are mentioned in the materials and method section. All expression were normalized with Arabidopsis *Actin2*. The data shown are the mean of three independent biological replicates ± SD. Student's t-test (*) P < 0.5, (**) P < 0.05, (***) P < 0.005, related to the control. C, Control and T, Treated

We wanted to understand the expression profile of *Arabidopsis* VDACs under different stress treatment. Therefore, we performed quantitative real-time PCR to analyse the expression of the whole *VDAC* gene family in *Arabidopsis thaliana*. The *Arabidopsis* Col-0 plants were subjected to abiotic stresses such as cold, salt, ABA, and drought; and biotic stress by using Pst (DC3000); and MV treatment. In the control (0 h samples) *VDAC1* and *VDAC2* were relatively highly expressed than *VDAC3* and *VDAC4*. This situation was also reflected in the stress treated samples of cold, ABA and drought. Under these treatments, the *VDAC1* and *VDAC2* transcripts again dominated *VDAC3* and *VDAC4*. Surprisingly under salt treatment, all the transcripts showed lower expression than control. In our MV stress treatment, we observed that *VDAC2* and *VDAC4* were upregulated than control, especially *VDAC4,* which was highly upregulated. Our Pst (DC3000) challenge showed that with the increase of the treatment time (12 h), *VDAC1*, *VDAC2*, and *VDAC4* transcripts were upregulated compared to control. All this information is summarized in **Figure 1**. The expression pattern of the *Arabidopsis VDAC* gene family from publicly available microarray data (eFP browser, www.bar.utoronto.ca) is represented in **Supplementary Figures S1A** (abiotic stress), **S1B** (biotic stress), and **S1C** (development). This data indicates that VDACs are generally upregulated when challenged with pathogens.

### *Arabidopsis* Voltage-Dependent Anion Channels Can Restore the Temperature Dependent Growth Defect of *Δpor1*

**Figure 2:**
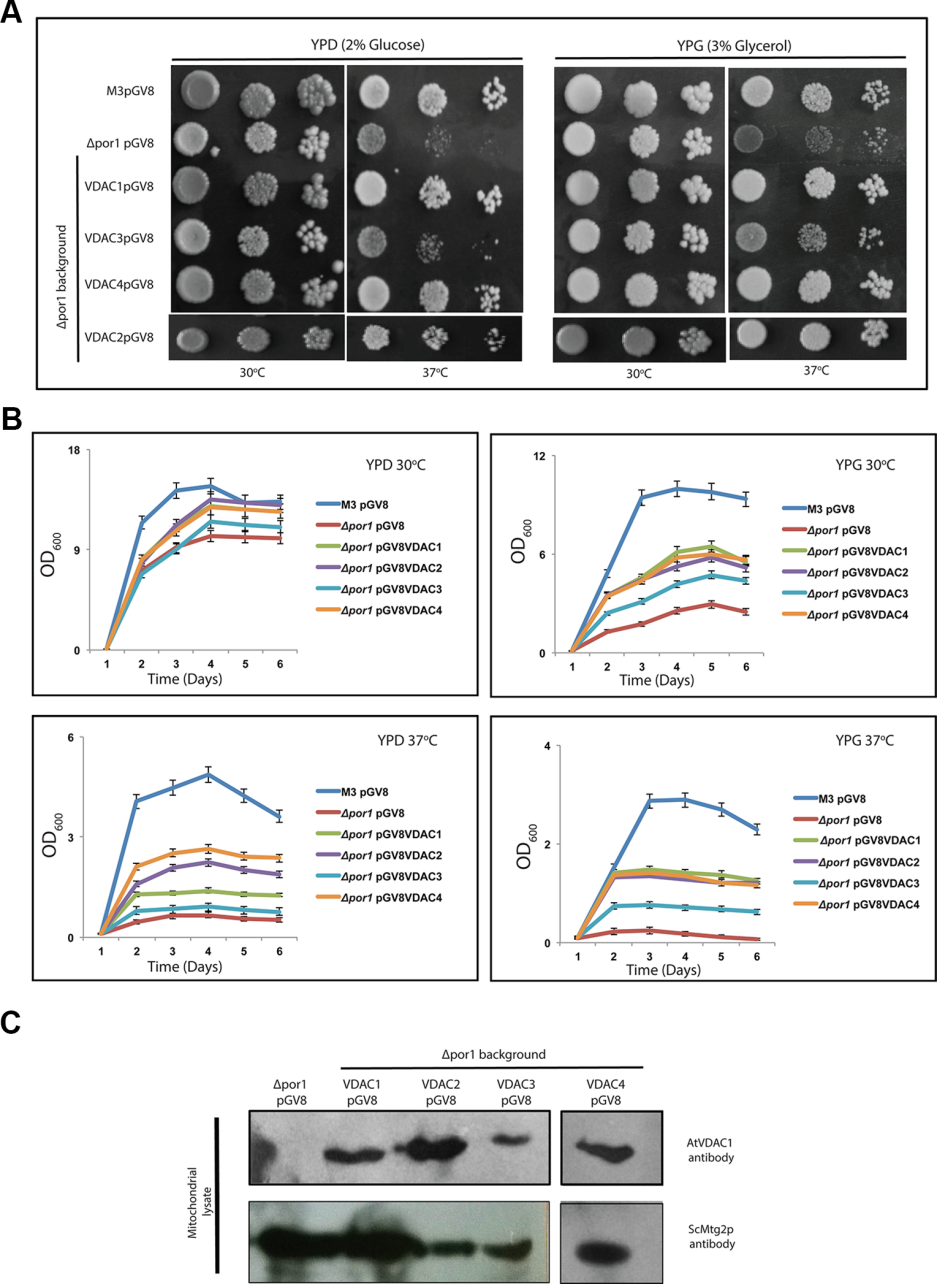
*Arabidopsis* VDACs differentially complement *Δpor1* yeast in complementation assay. **(A)** *Δpor1* transformed with different VDACs were tested in a drop-serial dilution assay. Cells were plated on media containing 2% glucose (YPD) or 3% glycerol (YPG) as the sole carbon source and incubated at 30°C and 37°C for 5 days and then their growth was documented. *VDAC1, VDAC2* and *VDAC4* enable *Δpor1* to grow on glucose and glycerol at 37°C. A partial recovery is also observed in the case of *VDAC3*. **(B)** Growth kinetics of the *Δpor1* transformed with different VDACs in YPD and YPG at different temperatures. The data shown are the mean of three independent biological replicates ± SD. **(C)** Western blot analysis of mitochondrial lysates of *Δpor1* yeast strain expressing (empty) pGV8 and *Arabidopsis VDAC1, VDAC2, VDAC3*, and *VDAC4*. The antibody anti-*AtVDAC*1 (1:5,000, Agrisera) was used to identify VDAC1, VDAC2, VDAC3, and VDAC4 and anti-Mtg2p (1:2,000) yeast antibody was used as a mitochondrial marker. *Arabidopsis* VDAC1, VDAC2, VDAC3, and VDAC4 proteins were expressed and directed to mitochondria of *Δpor1* yeast cells.

*ScVDAC1* lacking yeast (Δpor1) shows a growth defect when grown on non-fermentable carbon source (like glycerol) at elevated temperature. This growth defect at elevated temperature is seen even when a fermentable carbon source is used. To analyze the effect of *Arabidopsis* VDAC complementation on yeast growth, yeast strains were transformed with the pGV8 construct carrying the respective *Arabidopsis* VDAC isoforms, or with the empty vector as a control. The growth pattern revealed that the complementation of *Δpor1* by *VDAC1, VDAC2* and *VDAC4* rescued it at a higher temperature (37°C) in both YPD and YPG media (**Figure 2A**). In the case of *VDAC3*, the complementation was much subtle in comparison to *VDAC1*, *VDAC2*, and *VDAC4* (**Figure 2A**). We corroborated this quantitatively by performing an optical density based growth kinetics assay in liquid culture using both YPD and YPG at 30 and 37°C. The results again indicated that *VDAC1, VDAC2* and *VDAC4* complementation could improve *Δpor1* growth at elevated temperatures and *VDAC3* complementation resulted in subtle growth improvement (**Figure 2B**). Immunoblot analysis revealed that all VDACs (VDAC1, VDAC2, VDAC3, and VDAC4) were correctly expressed in *Δpor1* strains and targeted to the mitochondria. **Figure 2C** shows a western blot of proteins extracted from mitochondrial lysates of Δ*por1* cells transformed with *Arabidopsis* VDACs. These results indicate that the VDAC proteins are expressed in yeast and correctly targeted to the mitochondria. The antibody (anti *Arabidopsis* VDAC1, Agrisera) used for western blotting could not detect the ScVDAC (1 or 2) proteins. The anti-Mtg2p was used as a mitochondrial marker (Datta et al., 2005) and loading control.

**Figure 3:**
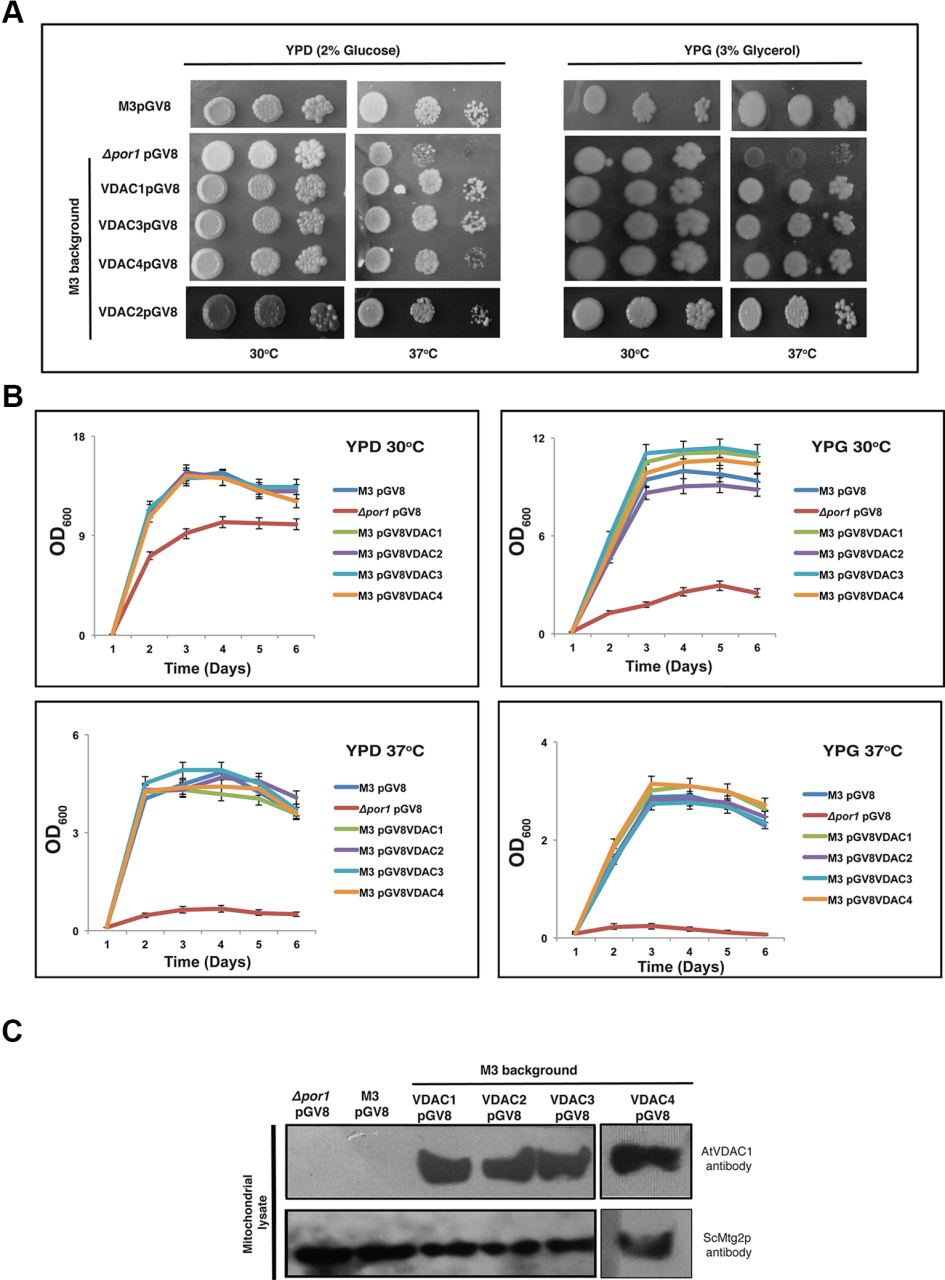
Expression of *Arabidopsis* VDACs in wild type M3 cells. **(A)** M3 transformed with different VDACs were tested in a drop-serial dilution assay. Cells were plated on media containing 2% glucose (YPD) or 3% glycerol (YPG) as the sole carbon source and incubated at 30 and 37°C for 5 days and then their growth was documented. **(B)** Growth kinetics of the wild type *M3* transformed with different VDACs in YPD and YPG at different temperatures. The data shown are the mean of three independent biological replicates ± SD. **(C)** Western blot analysis of mitochondrial lysates of M3 yeast strain expressing (empty) pGV8 and *Arabidopsis VDAC1, VDAC2, VDAC3*, and *VDAC4*. The antibody anti-*AtVDAC*1 (1:5000, Agrisera) was used to identify VDAC1, VDAC2, VDAC3, and VDAC4 and anti-Mtg2p (1:2,000) yeast antibody was used as a mitochondrial marker. *Arabidopsis* VDAC1, VDAC2, VDAC3, and VDAC4 proteins were expressed and directed to mitochondria of M3 yeast cells.

The overexpression of *VDAC1*, *VDAC2*, *VDAC3*, and *VDAC4* in M3 did not bring about any marked difference in the M3 phenotype at a higher temperature in both YPD and YPG media (**Figure 3A**). To corroborate this quantitatively, we again performed an optical density based growth kinetics assay in liquid culture using both YPD and YPG at 30 and 37°C. The results once again indicated that *VDAC1, VDAC2, VDAC3*, and *VDAC4* overexpression did not significantly change the growth pattern of M3 (**Figure 3B**). Immunoblot analysis revealed that all VDACs (VDAC1, VDAC2, VDAC3, and VDAC4) were correctly expressed in the mitochondria of M3 strains (**Figure 3C**).

### The Mitochondrial Membrane Potential and Reactive Oxygen Species Homeostasis Is Restored in Yeast Due to *Arabidopsis* Voltage-Dependent Anion Channel Expression

**Figure 4:**
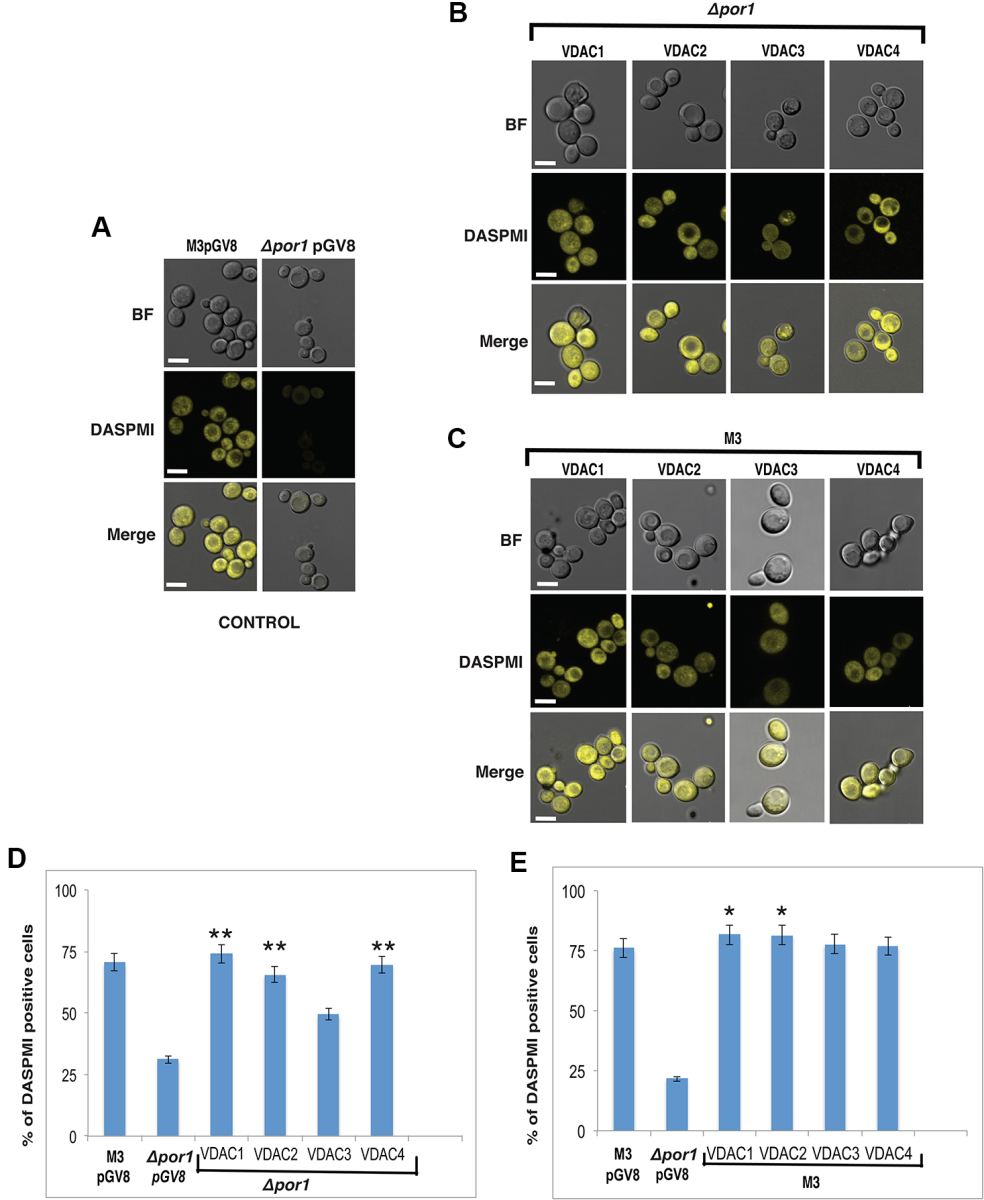
Effect on MMP due to complementation and overexpression of VDACs. **(A)** Confocal microscopy of M3 and *Δpor1* cells. M3 shows higher uptake of DASPMI than *Δpor1*. White scale bar in 5 μm. **(B)** Confocal microscopy of *Δpor1* cells complemented with VDACs. White scale bar in 5 μm. **(C)** Confocal microscopy of M3 cells overexpressing VDACs. White scale bar in 5 μm. **(D)** MMP measurement in *Δpor1* cells transformed with VDAC (1, 2, 3, and 4) grown in 3% YPG by flow cytometry. The *Δpor1* shows lower DASPMI positive cells than M3 when grown in YPG. Complementation of VDACs (1, 2, 3, and 4) in Δpor1 results in higher DASPMI positive cells restoring the mitochondrial health. The data shown are the mean of three independent biological replicates ± SD. Student's t-test (**) P < 0.001 related to the *Δpor1* pGV8. **(E)** MMP measurement in M3 cells transformed with VDAC (1, 2, 3, and 4) grown in 3% YPG by flow cytometry. Overexpression of VDACs (1, 2, 3, and 4) in M3 further increases the DASPMI positive cells. The data shown are the mean of three independent biological replicates ± SD. Student's t-test (*) P < 0.001 related to the M3pGV8.

The mitochondria help in generating ATP to power the cell. The MMP is an important physiological parameter that relates to the cells' capacity to generate ATP (Perry et al., 2011). DASPMI dye, whose uptake in cells is MMP dependent, has been previously used to measure MMP using microscopy and flow cytometry (Reina et al., 2010; Magri et al., 2016). We used YPG as the culture medium over YPD for our experiments to strictly maintain a respiration-based environment (Galganska et al., 2010a). Using confocal microscopy, we observed that a higher number of M3 cells showed DASPMI fluorescence than *Δpor1* corroborating with earlier results [**Figure 4A** and (Reina et al., 2010)]. On further investigation of the complemented cells, we found that *VDAC1* to *VDAC4* expressing *Δpor1* cells had their MMP restored (inferred by the DASPMI uptake by these transformants) (**Figure 4B**). To investigate the result quantitatively, we used flow cytometry, which has been recently used to measure MMP in the same system (Magri et al., 2016). Our results showed that M3 had a higher MMP as compared to *Δpor1* cells (about threefold more DASPMI positive cells) agreeing with the published results [**Figure 4D** and (Magri et al., 2016)]. The complementation of *Δpor1* with *VDAC1*, *VDAC2*, *VDAC3*, and *VDAC4* reversed the trend from a non-complemented *Δpor1* cell having an MMP similar to M3 (roughly about 2-3 fold more DASPMI positive cells) (**Figure 4D**).

**Figure 5:**
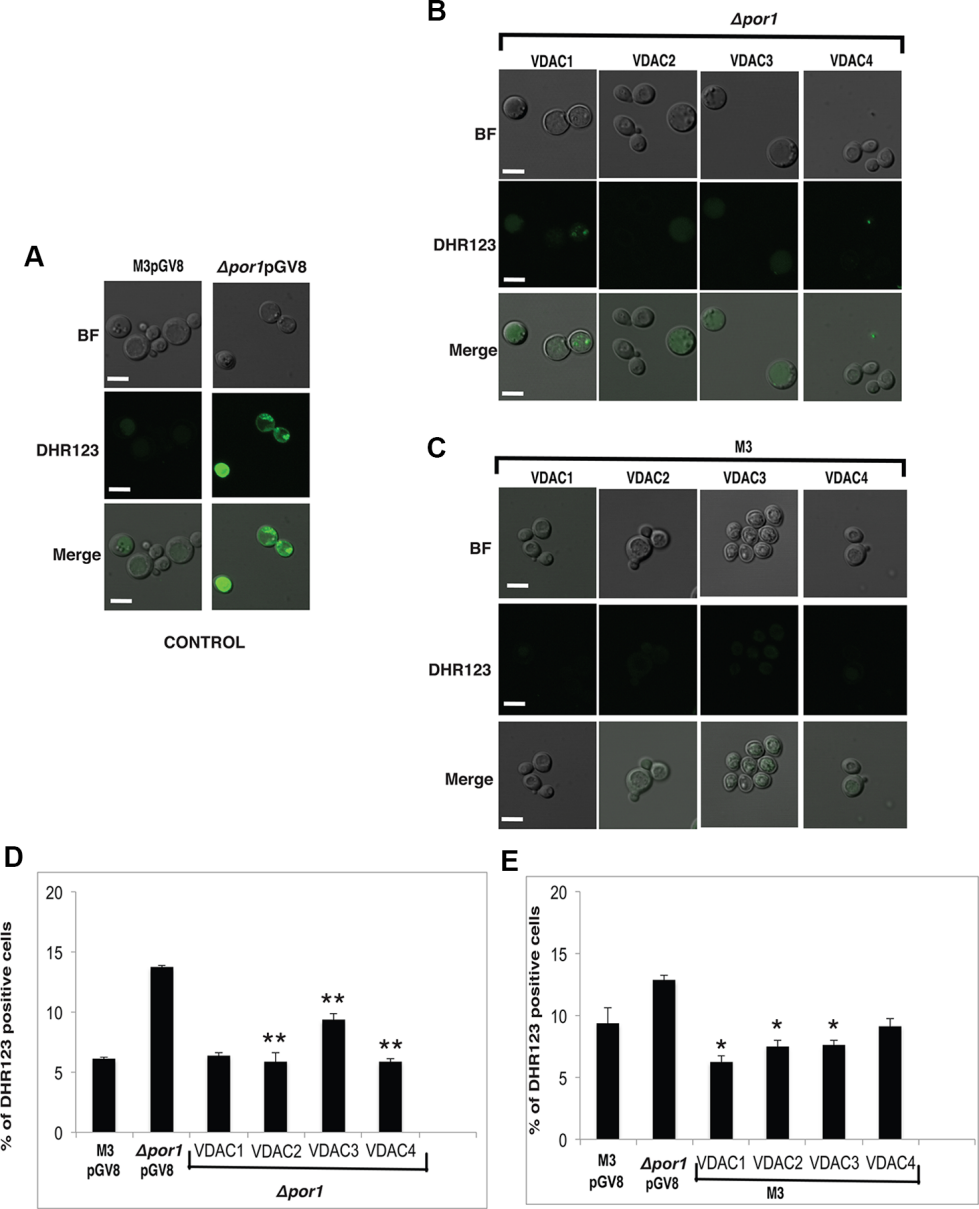
Reactive oxygen species (ROS) measurement in yeast samples. **(A)** Confocal microscopy of M3 and *Δpor1* cells. *Δpor1* shows higher uptake of DHR123 than M3. White scale bar in 5 μm. **(B)** Confocal microscopy of *Δpor1* cells complemented with VDACs. White scale bar in 5 μm. **(C)** Confocal microscopy of M3 cells overexpressing VDACs. White scale bar in 5 μm. **(D)** Total ROS measurement in *Δpor1* cells transformed with VDAC (1, 2, 3, and 4) grown in 3% YPG. The *Δpor1* shows a higher ROS content than M3 when grown in YPG, probably due to poorly active mitochondria. Complementation of VDACs (1, 2, 3, and 4) in *Δpor1* lowers the ROS level. The data shown are the mean of three independent biological replicates ± SD. Student's t-test (**) P < 0.001 related to the *Δpor1* pGV8. **(E)** Total ROS measurement in M3 cells transformed with VDAC (1, 2, 3, and 4) grown in 3% YPG. Overexpression of VDAC (1, 2, 3, and 4) in M3 further lowers the ROS level. The data shown are the mean of three independent biological replicates ± SD. Student's t-test (*) P < 0.001 related to the M3pGV8.

Superoxide anions (a class of ROS) are produced during respiration and functional mitochondria would maintain a proper balance of these in the cell. To analyze the functioning of the VDACs during respiration, total ROS content in transformed yeast growing in YPG media was estimated quantitatively and qualitatively using microscopy and flow cytometry, and measuring the fluorescence emission of rhodamine, the oxidation product of DHR123 (Reina et al., 2010; Magri et al., 2016). YPG was again preferred over YPD to strictly maintain a respiration-based environment (Galganska et al., 2010a). The *Δpor1* had more number of DHR123 fluorescing cells as compared to M3 in this condition (**Figure 5A** control). Agreeing with the MMP results, *VDAC1*, *VDAC2*, *VDAC3*, and *VDAC4* complemented cells showed lower DHR123 fluorescing cells as compared to *Δpor1* (**Figure 5B**). Here again, we quantified the ROS positive cells using flow cytometry. In the complemented *Δpor1* cells (with *VDAC1, VDAC2, VDAC3* and *VDAC4*), the numbers of DHR123 positive cells were lower as compared to non-complemented *Δpor1* mutant (about 1.5-3 fold decrease) indicating that the ROS balance was restored in these cells. But in *VDAC3* complemented cells, these were comparatively higher (about 1.5 fold decrease) than *VDAC1*, *VDAC2*, and *VDAC4* (about 3 fold decrease) (**Figure 5D**).

The overexpression of *VDAC1*, *VDAC2*, *VDAC3*, and *VDAC4* in M3 cells did not have any visible effect on M3 DASPMI accumulation (**Figure 4C**) and on quantification showed slightly higher (about 0.1 to 0.4 fold) MMP than M3 (**Figure 4E**). Investigating DHR123 uptake, again there was no visible difference (**Figure 5C**) but quantifying the result by flow cytometry, we observed a reduction in the number of DHR123 positive cells as compared to M3 cells (roughly about 1 to 2 fold) (**Figure 5E**).

### The *Arabidopsis* Voltage-Dependent Anion Channels Play a Role in Oxidative and Salt Stresses

**Figure 6:**
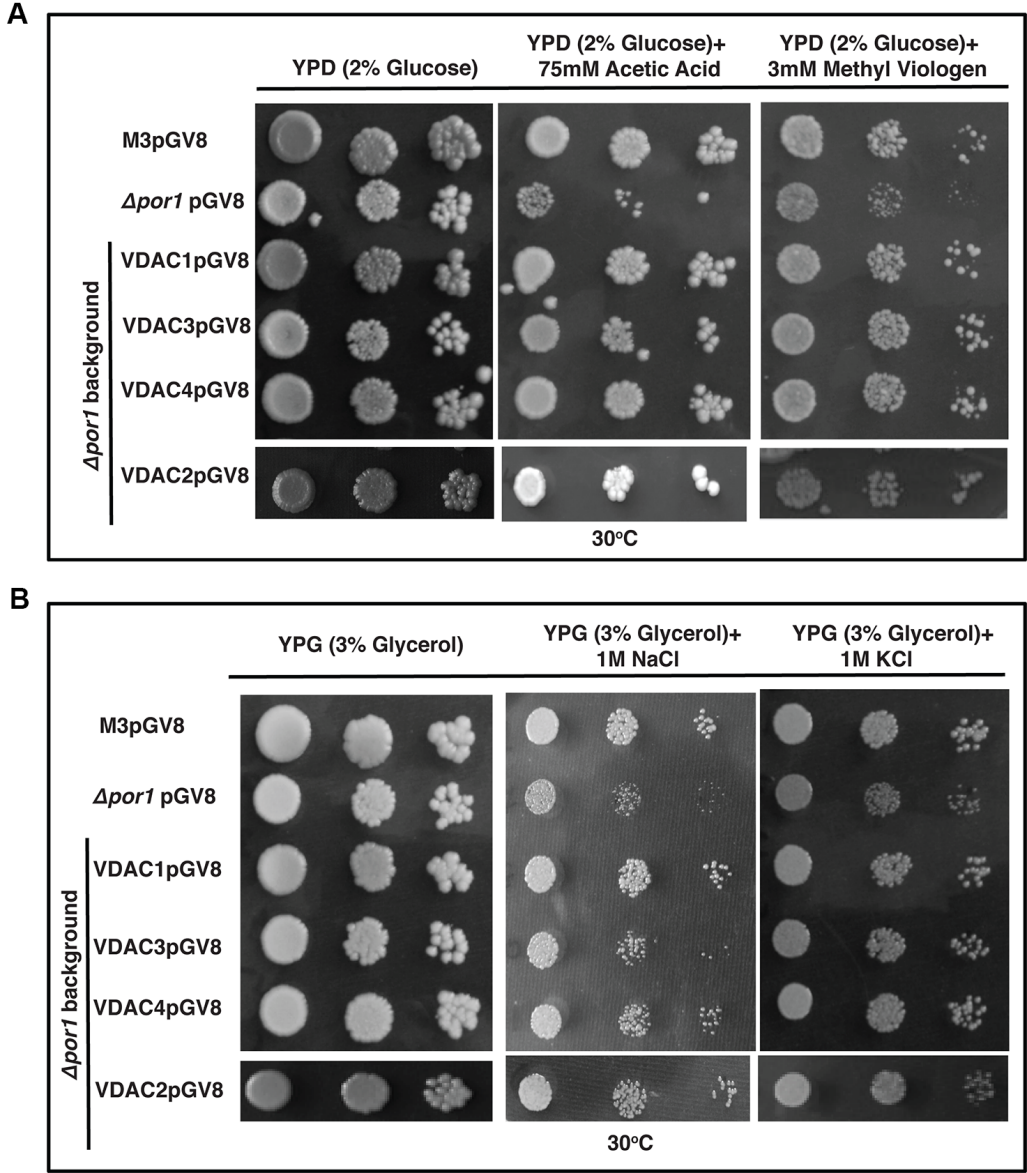
*Arabidopsis* VDACs complemented *Δpor1* cells survive in reactive oxygen species (ROS) and salt stresses. **(A)** *Δpor1* transformed cells with VDACs (1, 2, 3, and 4) survive reactive oxygen species generated during acetic acid and MV treatment. Cells were plated on media containing 2% glucose (YPD) with either 75mM acetic acid or 3mM MV. Plates were incubated at 30°C for 4 days and then documented. **(B)** *Δpor1* transformed cells with VDACs (1, 2, 3, and 4) were spotted on media containing 3% glycerol (YPG) with either 1 M NaCl or 1 M KCl. Plates were incubated at 30°C for 5 days and then documented. The controls in this figure are same as the controls in **Figure 2A**.

The *Δpor1* strain is susceptible to apoptosis by the ROS inducing agent acetic acid (Ludovico et al., 2001; Sanchez et al., 2001; De Pinto et al., 2010). Additionally, we used methyl viologen (MV) to concurrently check the effect of another ROS generator, which has a very different mode of action than acetic acid (Cocheme and Murphy, 2008; Magri et al., 2016). While acetic acid is an apoptogen for yeast, MV toxicity is due to the uptake of dication PQ^2+^ in the mitochondrial matrix and its subsequent conversion to a highly reactive radical PQ^.-^ (Ludovico et al., 2001; Cocheme and Murphy, 2008). The transformants were plated on YPD media containing either acetic acid or MV. *Δpor1* was highly sensitive in the presence of acetic acid as well as MV in the YPD medium and thus failed to grow (**Figure 6A**). In the case of acetic acid and MV, complementation with *VDAC1, VDAC2, VDAC3* and *VDAC4*, could rescue the mutant from oxidative stress (**Figure 6A**). The presence of high level of KCl and NaCl causes salt stress to the *Δpor1* mutant (Sanchez et al., 2001). To determine whether complementation by any of the VDACs confer tolerance to the *Δpor1* mutant to these agents, the transformants were screened on medium supplemented with either 1 M NaCl or 1 M KCl. As observed from (**Figure 6B**), *VDAC1*, *VDAC2,* and *VDAC4* rescued *Δpor1* mutant from salt stress thereby allowing it to grow on NaCl containing medium. In 1M KCl containing media, *VDAC3* performed similar to *VDAC1*, *VDAC2*, and *VDAC4* and complemented *Δpor1* cells were able to survive (**Figure 6B**).

**Figure 7:**
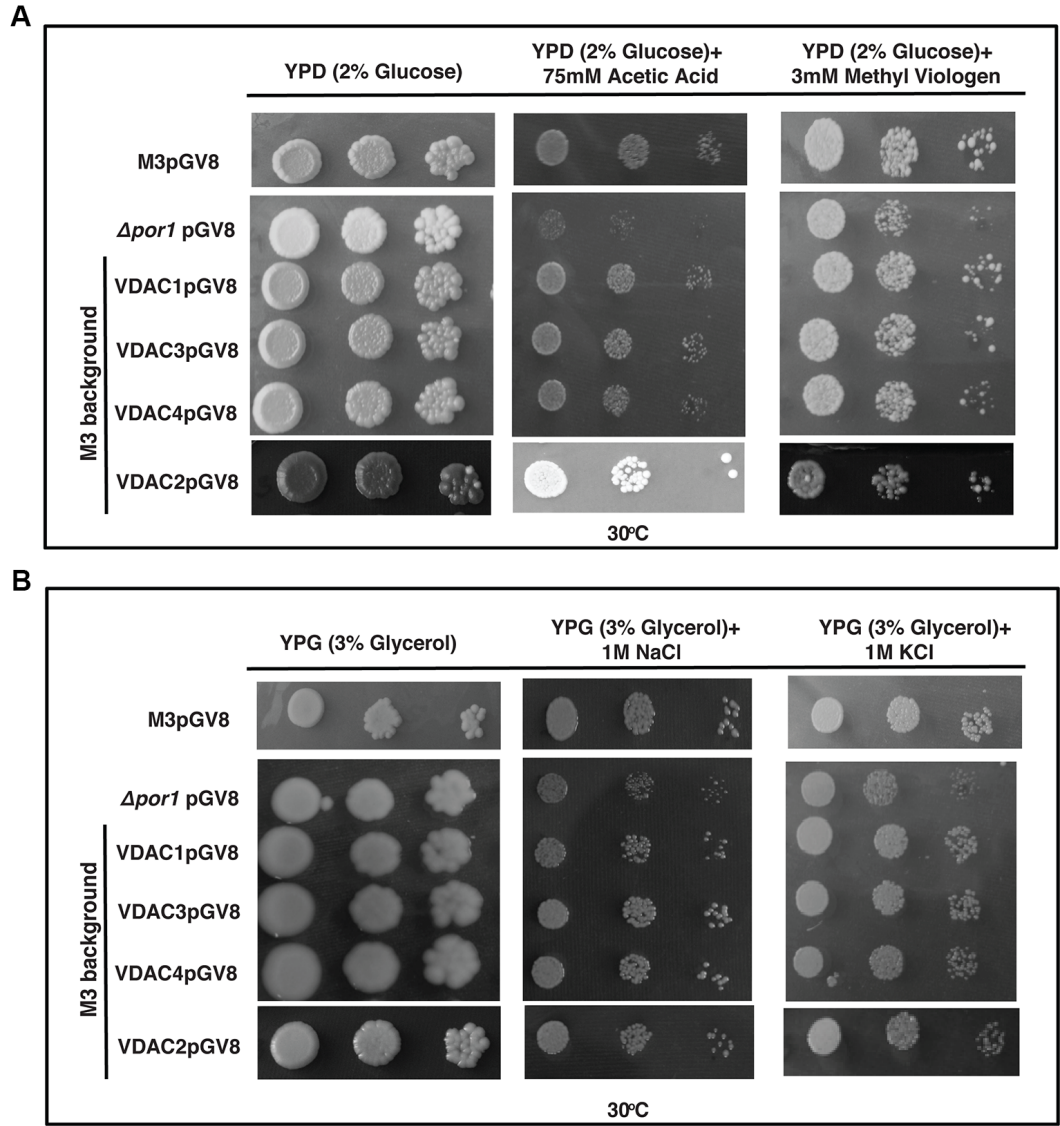
M3 cells subjected to oxidative and salt stresses. **(A)** M3 cells transformed with VDACs were spotted on media containing 2% glucose (YPD) with either 75 mM acetic acid or 3mM MV. Plates were incubated at 30°C for 4 days and then documented. None of the genes could affect (enhance/decrease) the growth of M3. **(B)** M3 cells transformed with different VDACs were spotted on media containing 3% glycerol (YPG) with either 1 M NaCl or 1 M KCl. Plates were incubated at 30°C for 5 days and then documented. The controls in this figure are same as the controls in **Figure 3A**.

Over-expression of *VDAC1*, *VDAC2, VDAC3*, and *VDAC4* caused no enhancement in the survival of M3 in the stress conditions used in this study (**Figure 7A**). Investigating the effect of salt stress in these cells revealed that *VDAC1*, *VDAC2*, *VDAC3*, and *VDAC4* overexpressing M3 cells survived both NaCl and KCl stress. (**Figure 7B**).

## Discussion

The *Saccharomyces cerevisiae* genome has two VDAC genes (*ScVDAC1* and *ScVDAC2*) (Blachly-Dyson et al., 1997). Seminal works on the ScVDAC mutants in yeast have proved that *ScVDAC1* null mutant yeast (*Δpor1)* shows a growth defect on glycerol media (Dihanich et al., 1987; Blachly-Dyson et al., 1997). However, the *ScVDAC2* loss-of-function does not show a similar growth defect (Blachly-Dyson et al., 1997). Recent reports from Kmita laboratory have shown the behaviour of the wild type yeast (with both the VDACs present), *Δpor1* (*ScVDAC1* mutant) and *Δpor2* (*ScVDAC2* mutant) grown in YPG in respiratory conditions (i.e., in glycerol) (Budzinska et al., 2007; Galganska et al., 2008; Budzinska et al., 2009). These collective reports suggest that *Δpor1* has a reduced ySOD1 (Cu/Zn Superoxide Dismutase) activity during exponential phase of growth resulting in the generation of higher O_2_^.-^ in the mitochondria, which are released in the cytosol *via* the Tom40 (translocase of the mitochondrial outer membrane) complex (Budzinska et al., 2007). In the case of wild type, in the exponential phase, a reverse case is seen and less O_2_^.-^ is released. The shift to stationary phase from exponential phase makes *Δpor1* cytosol more reduced and wild type cytosol more oxidized. A similar shift in the oxidation and reduction state is seen inside the mitochondria as well and the collective information has pointed towards the involvement of VDACs in regulating the redox state of the cell (Galganska et al., 2008).

The expression of *Arabidopsis VDACs* under different stimuli have been previously analysed in two different reports. While Lee and colleagues reported that *VDACs* are highly upregulated after Pst (DC3000) challenge compared to abiotic stress (Lee et al., 2009), Tateda and colleagues reported that both virulent (Pst (DC3000)) and avirulent (Pst DC3000 (*avrRpt2*) pathogens cause the upregulation of *VDAC*, with the avirulent strain causing higher expression (Tateda et al., 2011). However, there were reports in the interim that supported the VDACs role during abiotic stress (Yang et al., 2011; Li et al., 2013). So we decided to reinvestigate the expression profile of At*VDAC*s and we also introduced the oxidative stress stimuli (mimicked by MV) as a new parameter. Our abiotic stress treatments were different from the Lee and colleague report and we could see that the cold, drought and ABA could induce VDACs (*VDAC1* and *VDAC2*). The expression of *VDAC1* increased with the duration of stress and the result is similar to the report of Li and colleagues (Li et al., 2013). *VDAC2* also had enhanced expression under cold stress. In comparison, *VDAC3* and *VDAC4* have did not have a significantly higher expression in our assays. *VDAC3* (and its interactors *KP1*) has been previously implicated to be responsible for regulating seed germination at low temperature. As the mutants of *VDAC3* showed better germination in cold compared to wild type but the expression was not significantly perturbed by stress. We observed a similar trend for *VDAC4*. There are reports of VDACs being upregulated after drought stress or recovery after drought stress (Al Bitar et al., 2003; Desai et al., 2006). We found a similar upregulation for *VDAC1* and *VDAC2* indicating these two VDACs are upregulated and the other two *VDAC3* and *VDAC4* are not significantly upregulated. Surprisingly, our ABA treatment analysis did not exactly mimic drought treatment results. At 6 h point, the results were almost the same and *VDAC1* and *VDAC2* were upregulated, but prolonged treatment lowered the expression of all the isoforms. The downregulation of expression under ABA treatment has been previously reported for *VDAC2* (Yan et al., 2009) and so we can hypothesize that after a certain point, drought stress and ABA treatment have different effects on *VDAC* transcript, and the drastic fall is seen in the *VDAC* transcription at the 12 h point. Our MV treatment showed higher expression of *VDAC2* and *VDAC4* while the others were not upregulated and our biotic stress assay showed upregulation of all VDACs other than *VDAC3*. Taking all these results into perspective, we can hypothesize that VDACs could act as early response protein in stress and hence we see the upregulation. But all the transcripts are not upregulated together (in the majority of the stress) probably to save redundancy. It has been reported in the case of the human VDACs that each isoform can affect the expression of others to maintain co-ordination (De Pinto et al., 2010). So this factor may also play a role to regulate expression. Our salt stress analysis gave a contradictory result to our hypothesis. We observed that all the VDACs were downregulated under salt stress. Two other reports on *Arabidopsis* VDACs report either no change (Lee et al., 2009) or upregulation followed by downregulation of *VDAC3* in roots (Zhang et al., 2019). Apart from the difference in the treatment condition, which may have led to the difference in results, we can form a hypothesis based on *VDAC3*'s behaviour in salt stress. Zhang and colleagues report that the *VDAC3* overexpressing plants are sensitive to salt stress. Although they find the upregulation, it is followed by downregulation and plants may downregulate the transcripts (and corresponding protein). This may be a mechanism to stop more ROS from escaping into the cytosol through the VDAC channel. Another possibility, especially in case of salt stress, could be that some other transcriptional or post-translational mechanism might be controlling VDAC activity. It has been proved, in *S. cerevisiae, VDAC* has a role in maintaining the cytosolic redox state that regulates the expression of genes (Galganska et al., 2010b). So the salt stress mediated perturbation may lead to transcriptional regulation of VDAC transcripts. This led us to hypothesize that depending on the way a stimulus is administered to plant, it can result in a different transcriptional response.

The human VDACs have a notable role in ROS homeostasis (Reina et al., 2010). To the best of our knowledge, the *Arabidopsis* VDACs have not been studied together for this aspect. So we tested the expression profile of At*VDACs* under MV stress. We found that VDAC2 and VDAC4 are highly upregulated by MV indicating that these may have a role in ROS regulation (**Figure 1**). The earlier reports mention only the complementation capability of *Arabidopsis* VDACs, which we too have corroborated in our study (Lee et al., 2009). During our study, we have found that *VDAC3* could slightly complement the *Δpor1* mutant. A similar phenomenon was also reported for human VDAC3 where it similarly showed weaker complementation in glycerol and at elevated temperatures (De Pinto et al., 2010). The *Arabidopsis* VDACs were able to revive the MMP in *Δpor1* complemented cells to the level of wild type yeast indicating that in yeast they function to restore the ATP production mechanism. The measurement of cellular ROS during respiration confirmed that the *Arabidopsis* VDACs could lower the ROS content inside the mitochondria as compared to the *Δpor1,* corroborating the MMP results as a functional mitochondrion would maintain lower ROS during respiration. The susceptibility of the *Δpor1* cells to acetic acid and paraquat (MV) was abolished in the complemented lines. *VDAC3* during these treatments could complement to a level almost similar to the other VDACs. At this juncture, it was important to confirm if *VDAC3* has better complementation capability only during ROS stress. Hence we tested the response of the *VDAC3* complemented *Δpor1* mutant on high salt media, which would mimic salt stress. The difference in the complementation capabilities between *VDAC1, VADC2, VDAC4*, and *VDAC3* was evident in the NaCl stress treatment. The results of our study raise a pertinent question, how *VDAC3* can protect *Δpor1* cells from ROS stress similar to other VDACs whereas in other stresses (salt and growth in high temperature) it displays only subtle complementation? In a recent work from the Kmita laboratory, it was demonstrated that human *VDAC3* can interact with minocycline and protect the *Δpor1* cells from H_2_O_2_ when compared with human *VDAC1* and human *VDAC2* (Karachitos et al., 2016). We hypothesize that a similar functional role might be played by *Arabidopsis VDAC3* in managing the ROS mediated oxidative stress.

As a concurrent approach to investigate the effect of overexpression of VDACs, we expressed them in wild type strain, which has both VDAC genes i.e., *ScVDAC1* and *ScVDAC2*. Our results did not indicate any significant change in the phenotype under any stress. In an earlier report, it was shown that overexpression of human VDACs reduced the viability of the yeast cells and this was pointed towards the pro-apoptotic nature of the proteins (De Pinto et al., 2010). Also, the human VDACs have a higher number of Cys [VDAC1 (2 Cys), VDAC2 (9 Cys), and VDCA3 (6 Cys)] (De Pinto et al., 2016). A major function of the Cys is implicated in their role in redox sensitivity. Especially in the case of human VDAC3, ROS can irreversibly change the VDAC3 Cys oxidation state, which may result in the protein damage and/or modifying ROS level signaling to nearby proteins (De Pinto et al., 2016). The *Arabidopsis* VDACs in comparison have a lower number of Cys. Probably the lack/or a smaller number of Cys in *Arabidopsis* VDACs give them a unique way of functioning, which possibly makes the yeast cell overexpressing them tolerant to stress. Our MMP and ROS measurement experiments by DASPMI and DHR123, respectively showed that some of the VDAC overexpressing cells were performing slightly better than the wild type yeast. So, we hypothesize that the variation in the Cys residues might have some fundamental changes in the way plant and animal VDACs function.

## Conclusion

**Figure 8:**
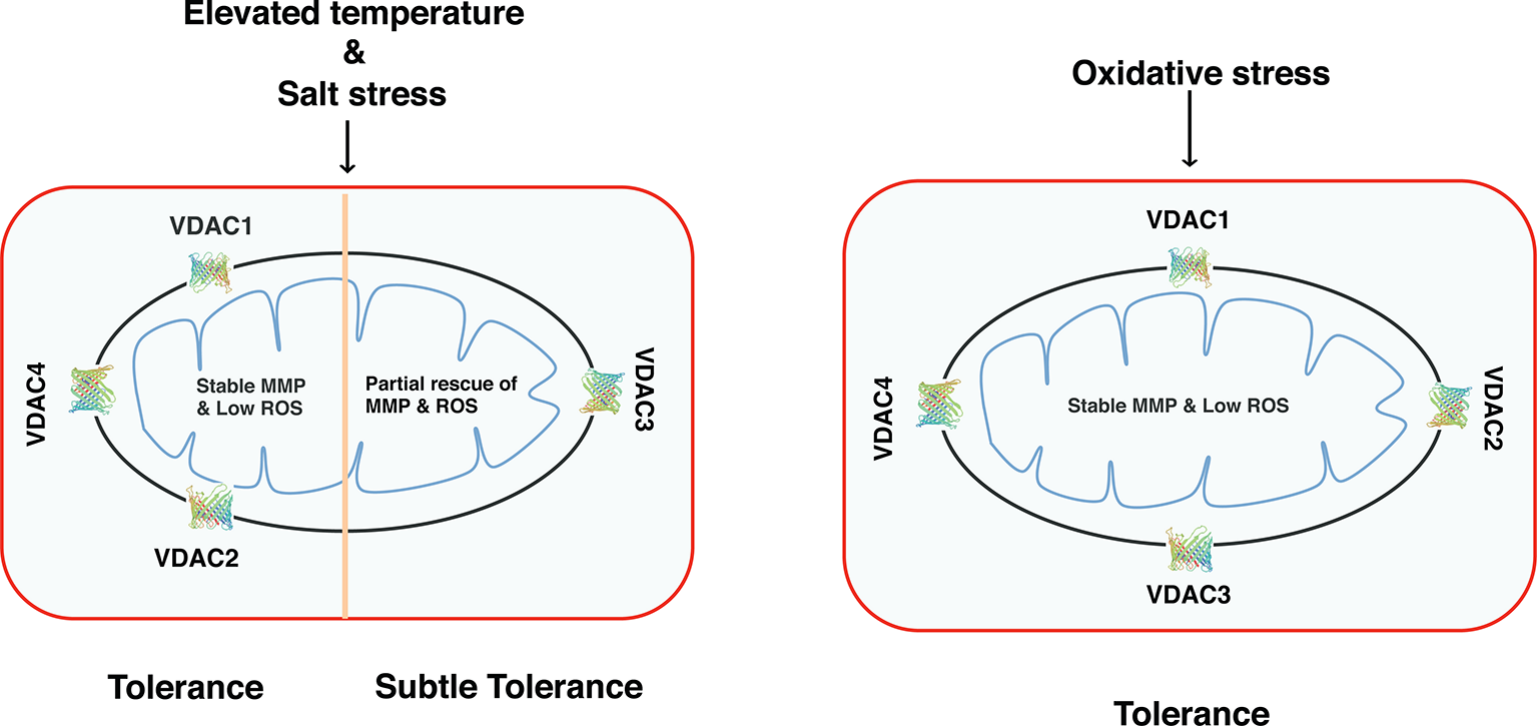
Functional diversity of *Arabidopsis* VDACs depending on their ability to complement *Δpor1*. VDAC1, VDAC2, and VDAC4 can complement the lack of *ScVDAC1* in *Δpor1* and maintain a stable mitochondrial membrane potential and also as a consequence maintain reactive oxygen species homeostasis. They can also survive under salt stress (as evidenced by our NaCl experiments). VDAC3 complemented *Δpor1* only show subtle complementation. When these transformants were analysed in oxidative stress (as evidenced by our acetic acid or methyl viologen tolerance experiments) the VDAC3 transformants show comparable results like the rest (VDAC1, VDAC2, and VDAC4) indicating a possible role of this protein in reactive oxygen species signaling/homeostasis.

Our results demonstrate that based on function, *Arabidopsis* VDACs could be divided into two groups (explained in a hypothetical model in **Figure 8**). Group 1 comprises of the VDAC1, VDAC2, and VDAC4, which can strongly restore respiration in *Δpor1* in elevated temperatures. These VDACs can also restore MMP and ROS homeostasis and protect the yeast mutant against salt and oxidative stress. Group 2 has VDAC3 and it can partially rescue *Δpor1* from growth defect in non-fermentable carbon source and salt stress. In oxidative stress, VDAC3 performs at par with the other VDACs indicating a more important role of this particular VDAC in ROS signaling/homeostasis. The homology modelling of *Arabidopsis* VDACs using the Zebrafish VDAC2 (4bum.1.A) as template showed that all of them are consistent with the *Zf*VDAC2 3D structure (**Supplementary Figure S2**). Another fact to consider is the post-translational modifications of VDAC in planta (Kerner et al., 2012). It has been reported that VDACs can be phosphorylated as well as acetylated in humans. This is another aspect that must be investigated to understand the regulation of VDACs. Further, in depth functional analysis of these *Arabidopsis* VDACs *in planta* will strengthen our experimental results.

## Data Availability Statement

The raw data supporting the conclusions of this article will be made available by the authors, without undue reservation, to any qualified researcher.

## Author Contributions

GP conceived and supervised the original research plan. SS devised and performed all the experiments. PK, AY, SM, and HS generated constructs and performed initial phenotypic assay. JF performed ROS and MMP detection and quantification assays. SS, AS, PS, and GP analysed the data. SS and GP wrote and revised the manuscript.

## Funding 

The research work in GP's laboratory is supported by grants from the Department of Atomic Energy (DAE), Board of Research in Nuclear Sciences (BRNS), Department of Biotechnology (DBT), Department of Science and Technology (DST-PURSE grant), Delhi University (R&D grant), India.

## Conflict of Interest

The authors declare that the research was conducted in the absence of any commercial or financial relationships that could be construed as a potential conflict of interest.

## Supplementary Material

Supplementary Table 1 List of real time qRT-PCR primers used in the study.

Supplementary Table 2 Cloning primers used in the study. Restriction sites are marked in red.

Supplemental Figure S1 Expression of the voltage-dependent anion channels gene family in *Arabidopsis eFP Browser*. Figure S1A depicts expression in abiotic stress, Figure S1B in biotic stress, and Figure S1C during plant development.

Supplemental Figure S2 The predicted putative 3D structure of *Arabidopsis* voltage-dependent anion channels. SWISS-MODEL and *Zf*VDAC2 X-ray 3-D structure (4bum.1.A) was used to predict the structures.
