# Supplementary material for: Arabidopsis Mitochondrial Voltage-Dependent Anion Channels Are Involved in Maintaining Reactive Oxygen Species Homeostasis, Oxidative and Salt Stress Tolerance in Yeast
Source: Front Plant Sci. 2020 Feb 28;11:50. doi: 10.3389/fpls.2020.00050 (PMC7058595; doi:10.3389/fpls.2020.00050)
Supplement: Supplementary Table 1 — List of real time qRT-PCR primers used in the study. [file DataSheet_1.docx]

**Supplementary Material**

**Supplementary Table 1:** List of real time qRT-PCR primers used in the study

| **Isoform Name** | **Primer Name** | **Sequence 5' → 3'** |
| --- | --- | --- |
| **VDAC1** | VDAC1RT F | AGCATGGGATTGACTCAGAA |
|  | VDAC1RT R | GGTGAAATTTCCAGACTTGG |
| **VDAC2** | VDAC2RT F | GCAATACTTCCACGACCAC |
|  | VDAC2RT R | AGCACCAAATGAGATGACTG |
| **VDAC3** | VDAC3RT F | TCTACCAGCGTTGGATTCAC |
|  | VDAC3RT R | TGCCAGATTCGGTGTTATAG |
| **VDAC4** | VDAC4RT F | ATGCAGGGATTGGTTTCAAC |
|  | VDAC4RT R | ATGTGGTTGGGTTCACTGTG |
| **VDAC5** | VDAC5RT F | TGGAGCTCTTCTGCAACAC |
|  | VDAC5RT R | AGCGAGAGACCTAACCTTG |
| **VDAC6** | VDAC6RT F | GGTGCTGAGGCGAAGTACAT |
|  | VDAC6RT R | AGAACCACCATTGCTCACACT |

**Supplementary Table 2:** Cloning primers used in the study

| **Constructs** | **Name** | **Sequence 5' → 3'** |
| --- | --- | --- |
| VDAC1pGV8 | VDAC1 F'BamHI | AGGATCCATGGTGAAAGGTCCCGGTC |
|  | VDAC1R EcoR1 | AGAATTCTCAAGGCTTGAGTGCGA |
| VDAC2pGV8 | VDAC2 F'BamHI | AGGATCCATGAGCAAAGGTCCAGG |
|  | VDAC2 R EcoR1 | AGAATCCTCAAGGTTTGAGAGCAAG |
| VDAC3pGV8 | VDAC3 F'BamHI | AGGATCCATGGTTAAAGGTCCAGG |
|  | VDAC3 R EcoR1 | AGAATTCTCAGGGCTTGAGAGCGAG |
| VDAC4pGV8 | VDAC4 F'BamHI | AGGATCCATGGGAAGCAGTCCAGCTC |
|  | VDAC4 R EcoR1 | AGAATTCTGGTTGAGGGCGAGGG |

Restriction sites are marked in red.
