# Supplementary figures and images for: Arabidopsis Mitochondrial Voltage-Dependent Anion Channels Are Involved in Maintaining Reactive Oxygen Species Homeostasis, Oxidative and Salt Stress Tolerance in Yeast
Source: Front Plant Sci. 2020 Feb 28;11:50. doi: 10.3389/fpls.2020.00050 (PMC7058595; doi:10.3389/fpls.2020.00050)

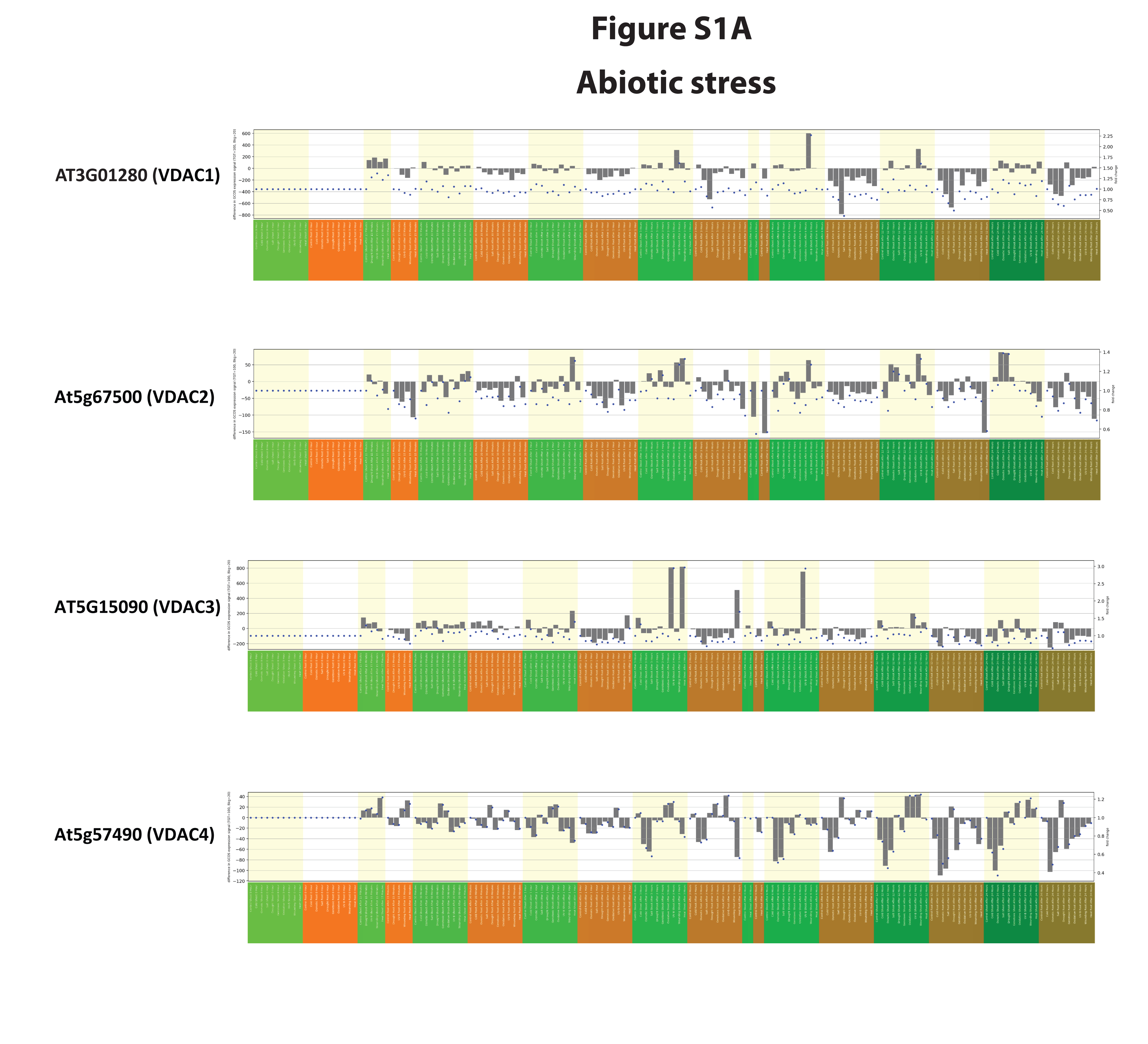

Supplement: Supplemental Figure S1 — Expression of the voltage-dependent anion channels gene family in Arabidopsis eFP Browser. Figure S1A depicts expression in abiotic stress, Figure S1B in biotic stress, and Figure S1C during plant development. [file Image_1.jpeg]

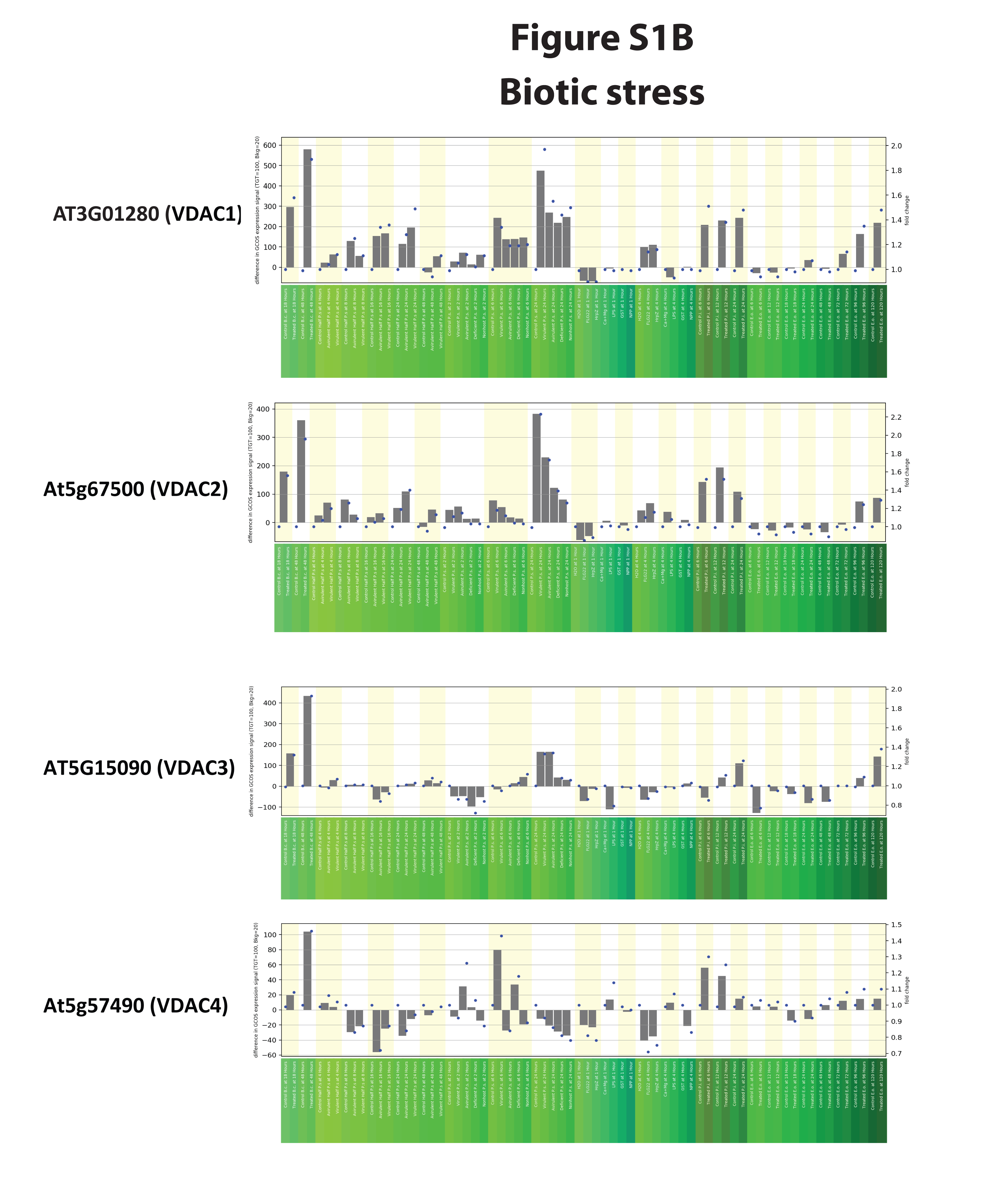

Supplement: Supplemental Figure S2 — The predicted putative 3D structure of Arabidopsis voltage-dependent anion channels. SWISS-MODEL and ZfVDAC2 X-ray 3-D structure (4bum.1.A) was used to predict the structures. [file Image_2.jpeg]

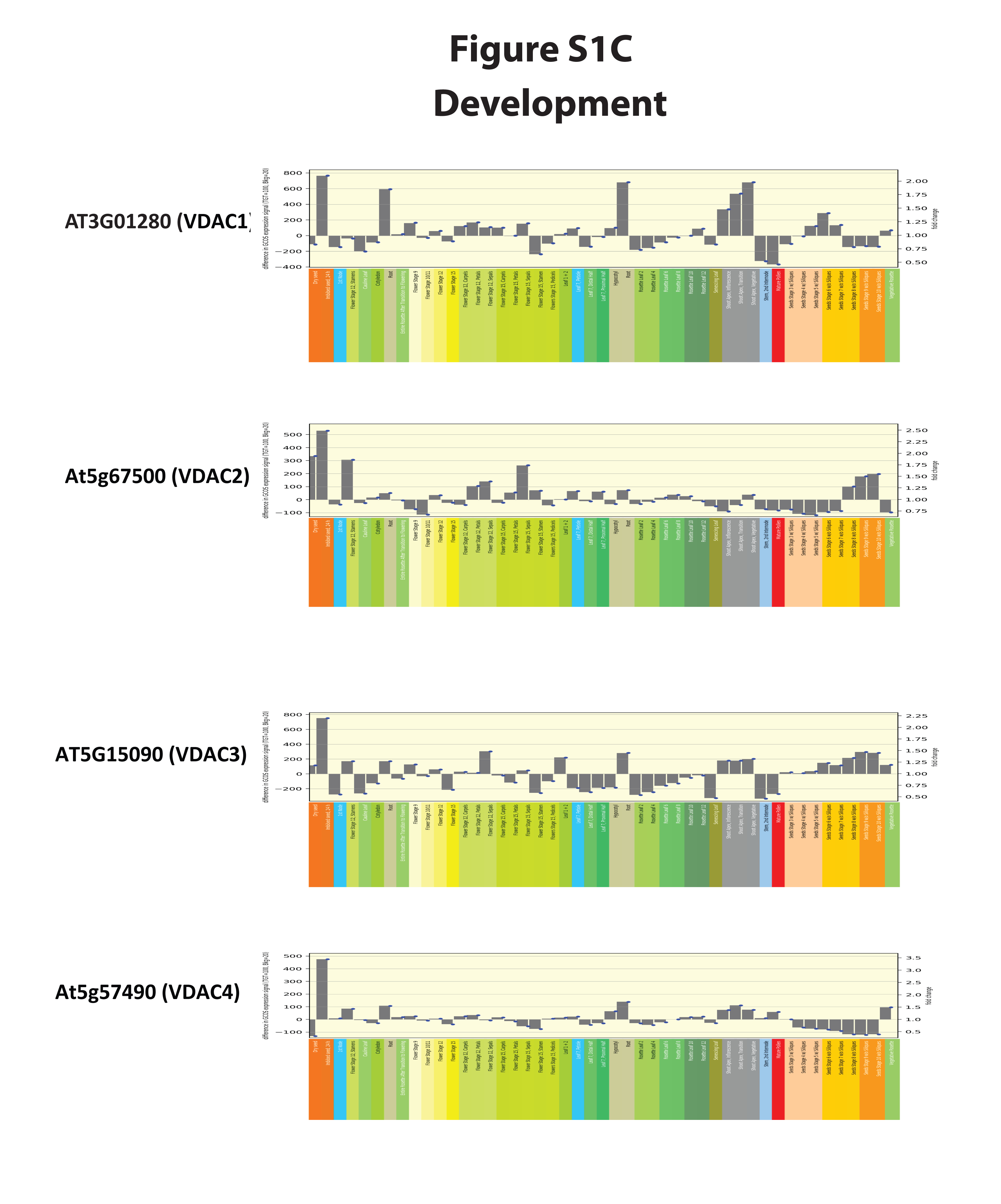

Supplement: Supplementary file 4 [file Image_3.jpeg]

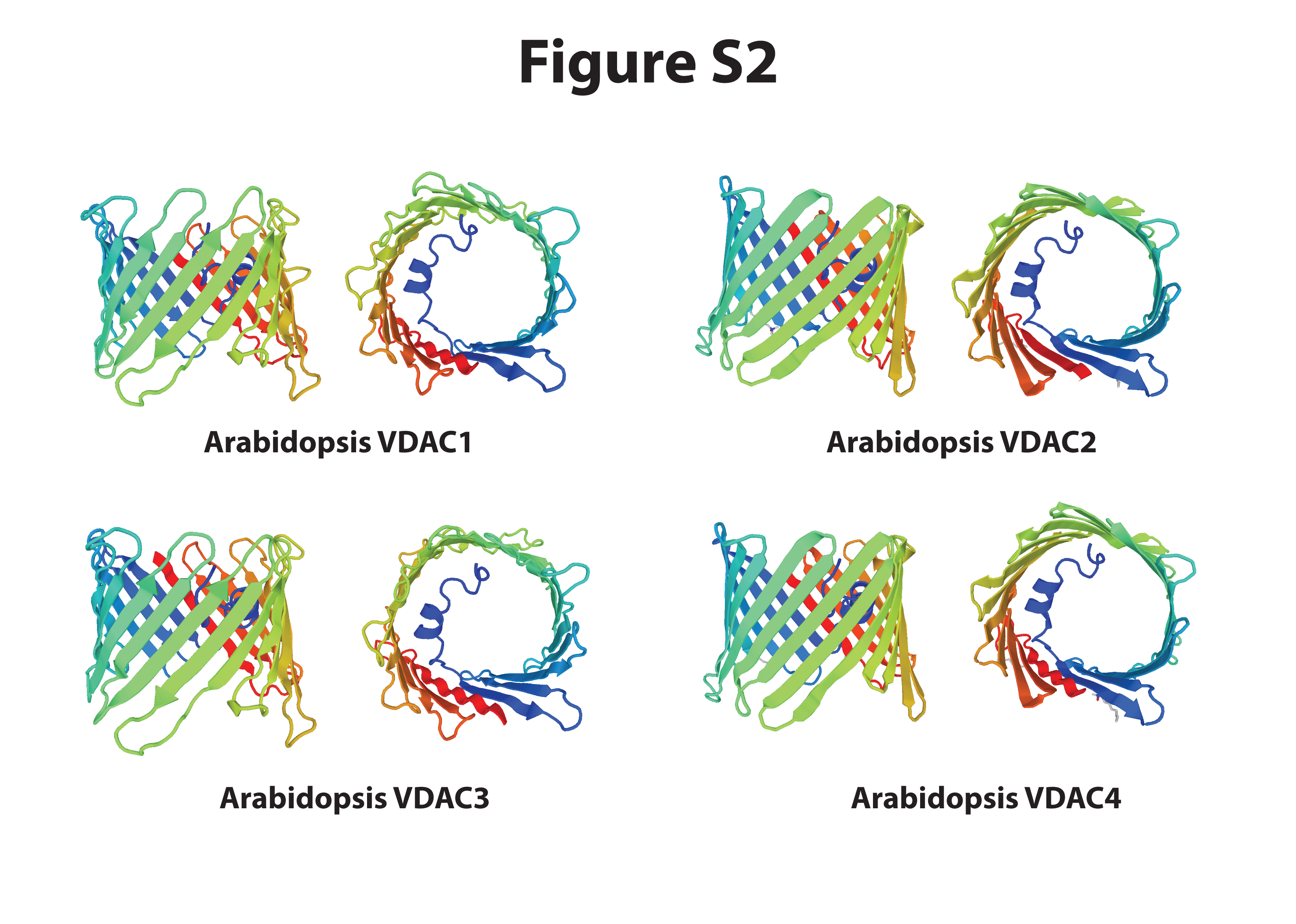

Supplement: Supplementary file 5 [file Image_4.jpeg]
